# New Structural Perspectives in G Protein-Coupled Receptor-Mediated Src Family Kinase Activation

**DOI:** 10.3390/ijms22126489

**Published:** 2021-06-17

**Authors:** Sandra Berndt, Ines Liebscher

**Affiliations:** Rudolf Schönheimer Institute of Biochemistry, Molecular Biochemistry, Medical Faculty, University of Leipzig, 04103 Leipzig, Germany; ines.liebscher@medizin.uni-leipzig.de

**Keywords:** G protein-coupled receptors, GPCR, SFK, Src kinases, G proteins, arrestin, allosteric regulation, biased signaling, non-receptor tyrosine kinases, SH3 domains, polyproline motifs, kinase activation, signaling

## Abstract

Src family kinases (SFKs) are key regulators of cell proliferation, differentiation, and survival. The expression of these non-receptor tyrosine kinases is strongly correlated with cancer development and tumor progression. Thus, this family of proteins serves as an attractive drug target. The activation of SFKs can occur via multiple signaling pathways, yet many of them are poorly understood. Here, we summarize the current knowledge on G protein-coupled receptor (GPCR)-mediated regulation of SFKs, which is of considerable interest because GPCRs are among the most widely used pharmaceutical targets. This type of activation can occur through a direct interaction between the two proteins or be allosterically regulated by arrestins and G proteins. We postulate that a rearrangement of binding motifs within the active conformation of arrestin-3 mediates Src regulation by comparison of available crystal structures. Therefore, we hypothesize a potentially different activation mechanism compared to arrestin-2. Furthermore, we discuss the probable direct regulation of SFK by GPCRs and investigate the intracellular domains of exemplary GPCRs with conserved polyproline binding motifs that might serve as scaffolding domains to allow such a direct interaction. Large intracellular domains in GPCRs are often understudied and, in general, not much is known of their contribution to different signaling pathways. The suggested direct interaction between a GPCR and a SFK could allow for a potential immediate allosteric regulation of SFKs by GPCRs and thereby unravel a novel mechanism of SFK signaling. This overview will help to identify new GPCR–SFK interactions, which could serve to explain biological functions or be used to modulate downstream effectors.

## 1. Introduction

Src family kinases (SFKs) are non-receptor protein-tyrosine kinases that regulate essential processes such as cell proliferation, differentiation, survival, migration, and metabolism [1].

SFKs are upregulated in malignancies, and their expression levels as well as specific activity are elevated in brain, breast, colon, lung, and pancreatic carcinomas [2,3,4,5,6,7,8,9]. For acute myeloid leukemia and colorectal cancer, a direct correlation between expression level of some SFK family members and patient survival was observed [10,11].

The human SFKs consist of eight typical family members (Src, Fyn, Yes, Fgr, Hck, Blk, Lck, and Lyn) and three atypical family members (Brk, Frk, Srms) based on sequence similarity. In some nomenclatures, atypical family members are considered a separate family also called the Frk family [12,13]. Src, Fyn, and Yes are expressed ubiquitously, while the other family members show tissue-specific expression [14]. Most of the SFKs, such as Yes, Fgr, Blk, Hck, Lck, and Lyn have an important regulatory role in signaling pathways of hematopoietic cells. A majority of SFKs are essential in immune response; whereas Fyn and Lck are activated immediately after T-cell receptor stimulation, expression of Fgr, Hck, Lyn is induced in stimulated mature monocytes and macrophages [15].

SFKs represent drug targets with great therapeutic potential, especially in cancer treatment, since SFKs are involved in cancer progression in various stages (reviewed in [16,17]). Approved drugs for cancer treatment targeting Src family kinases, such as Dasatinib, show a high level of toxicity [18] due to their unselective inhibition of SFKs in cancer and healthy cells. In-depth structural, functional, and mechanistic knowledge of each single SFK in combination with a detailed understanding of their expression regulation can be the basis for a more specific therapeutic approach with limited side effects.

## 2. Structural Hallmarks of SFKs

**Figure 1:**
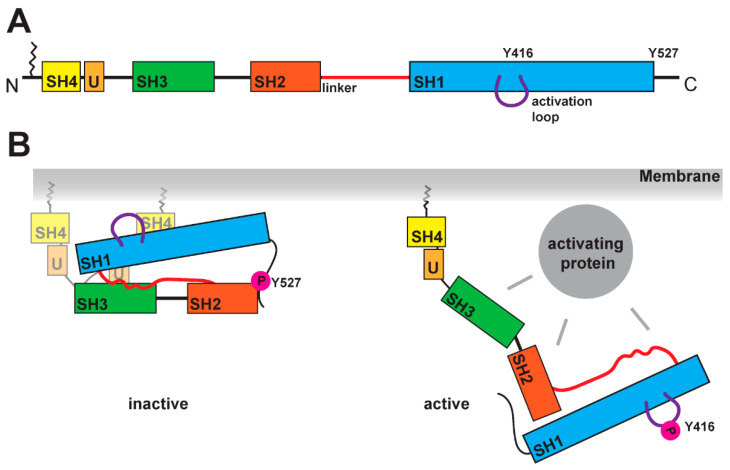
Schematic of SFK domains and activation mechanism. (**A**) SFKs are organized in several domains: the SH4 domain (in yellow), a unique domain (in light orange), the SH3 domain (in green), the SH2 domain (in dark orange), the SH1 domain (in blue). At the N-terminal end, SFKs contain a lipid anchor, which is localizing the kinase at the membrane. The linker region between the SH2 and SH1 domain is crucial for the activation mechanism. A further structural feature is the activation loop within the SH1 domain, which contains a tyrosine residue (for Src Y416) that is phosphorylated in the active state of the kinase. (**B**) Comparison of inactive and active states of SFK. In the inactive conformation, the tyrosine in the activation loop is not phosphorylated, while the tyrosine (Y527 for Src) at the C-terminus carries a phosphate residue as it binds to the SH2 domain. The closed conformation is stabilized by the linker region binding to the SH1 and SH3 domains simultaneously. SH4 and unique domain seem to be more flexible, and recent studies found binding of the SH4 domain to the SH1 domain [32] (two possible conformations are shown). The open conformation is induced by the binding of an activating protein, which can interact with the SH3, SH2, SH1 domains and the linker. This active conformation shows phosphorylation of the tyrosine (for Src Y416) in the activation loop and dephosphorylation of the tyrosine in the C-terminus (for Src 527).

SFKs are composed of distinct domains (Figure 1A). The N-terminal region, also called the SH4 domain, contains a myristoylation or palmitoylation site, which acts as a membrane anchor and is a key element for the localization of SFKs [19,20]. The unique domain, which is located after the SH4 domain, has a regulatory function for membrane localization and can form a fuzzy intramolecular complex with the neighboring SH3 domain [21,22,23]. SH3 domains serve as binding elements and are known to interact with a variety of polyproline motifs (reviewed in [24]). After a linker, SFKs contain the SH2 domain, known to interact with phosphorylated tyrosine residues, and following a longer linker region, the kinase domain, containing an N-lobe and a C-lobe. This domain entails two regulatory phosphorylation sites (Y-416 and Y-527 for Src as a representative example, Figure 1B) [25,26]. The first regulatory site is the activating autophosphorylation site, and the second one the negative regulatory site. The phosphorylation and dephosphorylation of these tyrosine residues cause dramatic structural changes and affect the activity of the kinase. In the inactive structure, Y-527 is phosphorylated by CSK (C-terminal Src kinase) or CHK (CSK homologous kinase) [27,28], which results in an interaction of the kinase domain with the SH2 domain [25,29,30]. This inactive conformation is further stabilized by the binding of the SH3 domain with the polyproline motif in the linker region between SH2 domain and kinase domain [30,31]. A recent finding showed a possible involvement of the SH4 domain, which binds in the inactive conformation to the kinase domain [32]. This compact state results in a closed conformation of the N- and C-lobes in the kinase domain, which results in a shielding of the Y-416 in the active site. In this closed conformation, the binding of ATP and substrates is blocked. In the active conformation, the interactions of SH2 and SH3 domains are displaced by other binding partners, which results in an open conformation (Figure 1B). This grants accessibility of the active site and allows for autophosphorylation of Y-416.

## 3. Modes of SFK Activation

In general, SFKs are activated by several different growth factor receptor tyrosine kinases. For example, the SH2 domains interact with SHP-1 protein tyrosine phosphatase, CRK-associated substrate, or protein tyrosine phosphatase-1B [33,34,35]. Proteins with typical polyproline motifs such as cyclin-dependent kinase-5, KCNB1, p21-acitvated kinase-2, vinculin and GRB2 have also been shown to induce the active conformation [36,37,38,39,40,41].

Additionally, for a number of G protein-coupled receptors (GPCRs), SFK activation was shown (Table 1). However, the exact activation mechanism of this interaction is poorly understood. It has been postulated that there are three ways of GPCR-mediated SFK activation: through arrestins, G proteins, or direct binding**.** A detailed understanding of the mechanism is highly desirable due to the potential druggability of GPCRs and the crucial role of SFKs in cancer development and progression.

## 4. Src Activation through a GPCR–Arrestin Complex

Until now, the best understood GPCR-mediated activation of SFK is arrestin-based. As early as 1999, arrestin-2-mediated Src activation by beta-2 adrenergic receptor (β_2_AR) stimulation was detected [63]. Later on, this was observed for multiple other receptors (Table 1). Until recently, there was no evidence of how this interaction could take place.

**Figure 2:**
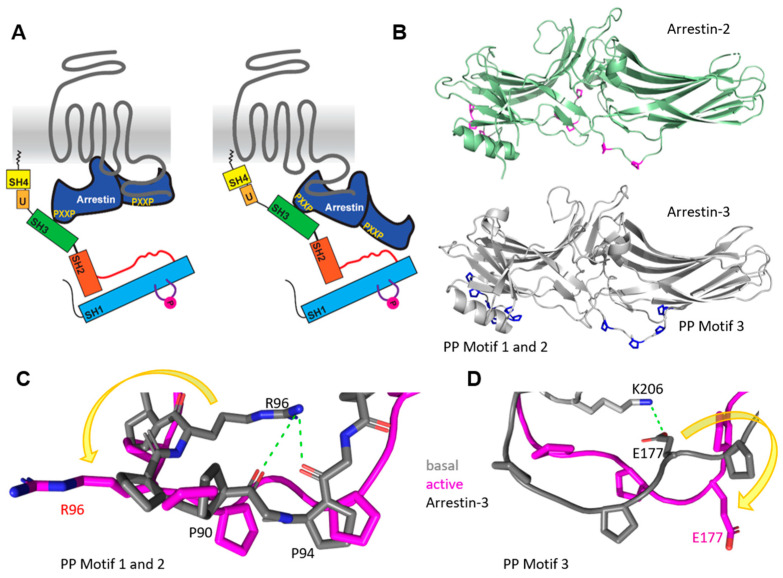
Binding motifs in arrestin-3 but not in arrestin-2 show structural rearrangement with activation. (**A**) Cartoon of arrestin-mediated SFK activation in the ‘core’ (**left**) and the ‘tail’ conformation (**right**) of arrestin. The activating receptor is shown in grey, arrestin is colored in dark blue with yellow polyproline motifs, and SFK color scheme was described earlier. (**B**) Structure of basal arrestin-2 in green with polyproline motifs in magenta (PDB file 1JSY [77]) and arrestin-3 with highlighted polyproline motifs in blue that are surface-accessible in a receptor-bound state (PDB file 3P2D [78]). (**C**) The comparison of polyproline motifs 1 and 2 in the basal (grey) and active (magenta) arrestin-3 conformations shows a large structural rearrangement with a 180° rotation of the R96 (indicated by a yellow arrow). In the basal state of arrestin-3, R96 forms electrostatic interactions with the backbone of the polyproline motif 1. (**D**) The comparison of polyproline motif 3 between basal (grey) and active (magenta) states shows an 180° outward movement of E177 in the active state (indicated by yellow arrow). For comparison of basal and active arrestin-3, PDB files 3P2D and 5TV1 were used [71,78].

Arrestins have two major functions in GPCR regulation. First, receptor desensitization and internalization through recruitment of clathrin-coated pits [64,65] and second, the recruitment and activation of effectors such as MAPK and SFKs ([63] and reviewed in [66,67]). However, the concept of purely arrestin-based signaling has been recently challenged [68]. The active state of arrestins can be induced through their binding to the phosphorylated C-terminus (‘tail’ conformation) or the hydrophobic intracellular pocket between the helices of a GPCR (‘core’ conformation) [69,70] (Figure 2A). Activation by other regulatory molecules such as IP6 or a C-tail phosphopeptide has also been described [71,72,73]. The ‘core’ conformation is essential for the desensitization of G-protein signaling, while arrestin in the ‘tail’ conformation loses its desensitization ability [74]. Nevertheless, in the ‘tail’ conformation, arrestin internalization and signaling are still possible. The most dominant conformational change during the activation of arrestin is the rotation of the N- and C-domains towards each other. With this domain rotation, multiple small conformational changes appear (also called switch regions for arrestin-3) [75]. It is predicted that at least one of the previously described switch regions in arrestin-3 could be unique for this protein. This regulatory element contains a polyproline motif, which is a classical binding motif for SH3 domains. The interaction of SFK SH3 domains with polyproline motifs in arrestin are substantial for the activation of SFKs [76].

Yang et al showed that the receptor phospho-tail allosterically regulates the different conformations within the polyproline motifs in arrestin-2, which subsequently allows for the binding of the SFK SH3 domain. leading to the adoption of an open active conformation of the kinase [76]. A further recent study verified that receptor-bound arrestin-2, but not free arrestin-2, is able to activate Src [43]. Here, the binding of the receptor phospho-tail to arrestin-2 was shown to be sufficient to activate arrestin-2 and therefore Src (Figure 2A). There are only a few activation studies for arrestin-3-mediated Src activation. For example, in the case of the alpha 2 adrenergic receptor, arrestin-3 acts like a molecular switch, resulting in Src-mediated ERK activation [42]. For dopamine D1 receptor, activation of Src in the presence of arrestin-3 was shown [45].

Interestingly, for PAR-1 (protease-activated receptor-1), arrestin-3 showed opposite effects compared to arrestin-2 [79]. While arrestin-3 appeared to mediate the degradation of Src with the activation of PAR-1, arrestin-2 was crucial for Src activation. Arrestin-2 and -3 have each three polyproline motifs, PXXP, that differ slightly (^88^**P**PA**P**, ^121^**P**NL**P** and ^178^**P**ER**P** for arrestin-2 and ^89^**P**PV**P**, ^94^P**P**RP**P**T**R**, ^175^**P**EK**P** for arrestin-3, Figure 2B). Most the polyproline motifs do not contain a positively charged arginine, which could contribute to high-affinity binding of SFKs [80,81,82]. The exemptions are R180 in arrestin-2 and R96 and R100 in arrestin-3. By comparing the active and basal crystal structures of arrestin-3 (PDB 3P2D for basal and 5TV1 for active arrestin-3) [71,78], we found that in the basal structure of arrestin-3, R96 stabilizes the polyproline loop in a potential inactive conformation through electrostatic interactions with the backbone of the amid bond of P92 and N93 (Figure 2C). Polyproline motif 3 indicated a similar stabilization of the basal conformation by an electrostatic interaction between K206 and the highly conserved E177 (Figure 2D). By comparison with the active arrestin-3 structure, we found that the side chains of R96 (Figure 2C) and E177 (Figure 2D) are rotated 180° outward, which could allow the rearrangement of the polyproline motif. This structural reorganization of the polyproline binding motif of arrestin-3 might potentially have a regulatory effect on the SH3 domain interaction of SFKs with arrestin-3. Even though arrestin-2 harbors an arginine in motif 3 (R180), no electrostatic interactions were found by comparing crystal structures of basal and active arrestin-2 (PDB 1JSY for basal arrestin-2, 6UP7 and 6U1N for active arrestin-2) [77,83,84], which could significantly alter the polyproline motif conformation within the different activation stages. This could result in different affinities for SH3 domains and, therefore, explain the observed different roles of arrestin-2 and -3 in PAR1 activation.

## 5. Src Activation by G Proteins

G proteins (heterotrimeric guanine nucleotide-binding regulatory proteins) contain α, ß, and γ subunits. The α subunits can be classified into four families based upon sequence similarity: Gαs, Gαi, Gαq, and Gα12 [85]. The ß and γ subunits form a signaling complex due to their strong interaction. Agonist-bound GPCRs activate G proteins by facilitating the exchange of GDP to GTP at the α subunit. This active state causes the dissociation of the Gα subunit from the membrane-anchored ßγ subunit. Activation of SFKs by G proteins can be achieved through either the α subunit or the ßγ subunit**.** The interaction with the α subunit was shown by in vitro studies using Y-530-phosphorylated Src, with Gαs or Gαi resulting in the activation of Src. The interaction is believed to be mediated through the kinase domain of the SFK and the switch II region of the Gα subunit [86]. The described two switch regions in G proteins are defined regions crucial for binding of effectors such as Ras protein or adenylyl cyclase [87,88,89]. The activation of Src through the ßγ subunit was found for the CRF1 receptor by using a ßγ subunit inhibitor which caused downregulation of Src activation [90]. For carvedilol-stimulated β_1_ adrenergic receptor, Src-dependent ERK activation was shown [91]. Here, it was suggested that the activation of Src also involved the Gßγ subunits of the G protein, whereas this complex formation was arrestin-dependent [92].

Src, contrastingly, is able to phosphorylate Gα subunits in vitro, whereas the highest efficiency is shown for the GDP-bound inactive subunit. The two sites of phosphorylation are Y37 and Y377 [93,94], and both promote GTP hydrolysis. The different regulatory mechanisms by G protein phosphorylation are reviewed elsewhere [95]. In transducin, an additional phosphorylation site, Y142, was found [96]. Furthermore, Gßγ subunits are possibly phosphorylated, but it is not known if SFKs are involved. Overall, an arrestin-independent G protein-mediated activation of Src is still not fully understood and requires further investigation.

## 6. Src Family Kinases as Direct Effectors of GPCRs

**Figure 3:**
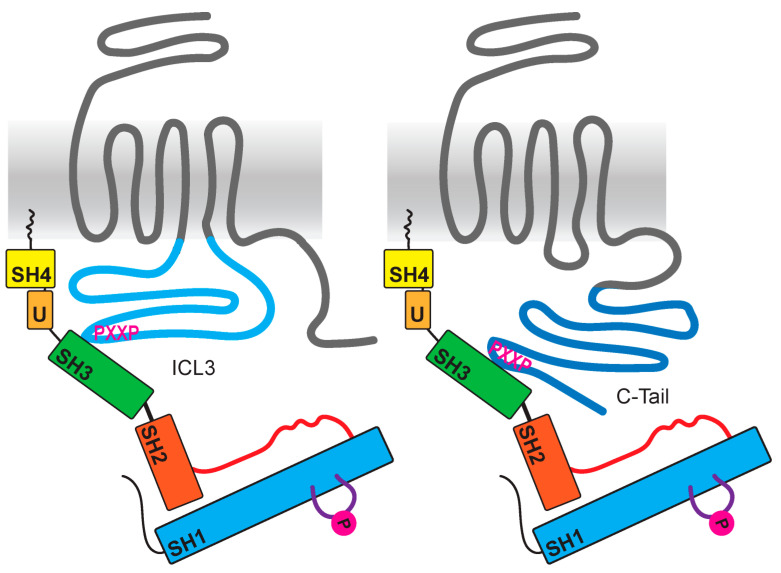
Direct interaction of GPCRs with SFKs through intracellular domains. Some GPCRs (colored in grey) encode polyproline motifs (shown in magenta) either in their third intracellular loop (light blue) or in their C-tail (colored in dark blue). For several receptors, an SFK SH3 domain (shown in green) interaction was verified.

The existence of protein binding motifs within the intracellular structures of GPCRs is well known; however, the impact on GPCR signaling remains poorly understood. A variety of binding motifs are located in the intracellular loops and C-termini for, e.g., PDZ proteins as well as SH2 or SH3 binding motifs [97]. Seventy-two out of 825 human GPCRs contain the classical polyproline SH3 domain-binding motif [76]. Most of these GPCRs have polyproline motifs within the third intracellular loop or the C-terminus (Figure 3), and for some of these receptors, an interaction with Src SH3 domains is predicted and was shown. For example, the beta-3 adrenergic receptor (β_3_AR) has typical SH3 binding sites in the third intracellular loop (Table 2), while it neither contains any GRK phosphorylation sites nor does it bind to arrestin [44]. β_3_AR mutations in Src binding sites inhibited the activation of Src or MAPKs. Nevertheless, β_3_AR Src activation is also Gαi-dependent. Further, the purinergic P2Y_2_ receptor entails polyproline motifs in the C-terminus, and Src binding, as well as its activation, was verified (Table 2) [98]. In most of these studies, the impact of arrestin was not taken into consideration.

Next to the typical interaction with polyproline motifs, another or an additional possibility is the interaction with phosphorylated tyrosine residues through the SH2 domain of SFKs. This could be shown for β_2_AR through mutation of residue Y350 in the C-terminal tail, which resulted in the decrease of Src phosphorylation and also impaired the desensitization of the receptor [99].

Dopamine receptors are a classical receptor family in which many family members contain polyproline motifs (Table 2). Multiple studies have confirmed the binding of SH3 domains to dopamine D2, D3, and D4 receptor [49,100,101]. However, it is unclear if there are additional adaptor or scaffold proteins involved in the activation mechanism. For dopamine D4 receptor, it could be shown that it directly activates the Src/SHC/Ras/ERK pathway [102]. The inhibition of Src by PP2 blocked ERK phosphorylation, which indicates signaling through Src for D2 and D4 receptors [103]. Recently, it was found that Fyn interacts with serotonin 5-HT6 receptor (5-HT6R) (Table 2) directly as well as in an arrestin-dependent manner to activate ERK1/2 [104].

## 7. GPCR-Mediated Src Signaling with Undefined Mediators

For several GPCRs, activation of an SFK was shown, but the exact regulation mechanism of the SFK is unknown. Some examples are the muscarinic M4 receptor (M4R), bradykinin receptor B1 (B1R), angiotensin type 2 receptor (ATR2), and A-type endothelin receptor (ETAR) (Table 1) [54,56,57,58,59,60]. For the V_1b_ vasopressin receptor (V_1b_R), Src activation was shown, and a potential interaction of the SH2 domain with intracellular domains of the receptor as well as an arrestin-mediated activation of Src was discussed [50]. Src activation was also shown for two adhesion GPCRs, Latrophilin-2 (ADGRL2) and GPR56 (ADGRG1) [61,62]. For Latrophilin-2, Src activation was observed, which could be either independent of or dependent on CDK5 [61]. For GPR56, overexpression of the receptor in 295T cells resulted in Src–Fak activation, which is RhoA-independent [62]. The C-terminus of Latrophilin-2 is exceptionally long, with 375 amino acids, which suggests that it could act as an adaptor for downstream effectors. GPR56, on the other hand, displays a rather short C-terminus, with only 35 amino acids. However, this C-terminus entails several potential phosphorylation sites, which hints at an arrestin-mediated activation of the Src-kinase.

In general, not many studies are available that address the direct interaction of GPCRs with SFKs and subsequent SFK activation. For a more detailed understanding, additional studies are needed to shed light on the multiple ways in which SFKs transduce GPCR-mediated signals. Similar to arrestin SFKs can provide an additional signaling option through a GPCR that contributes to the physiological roles of this receptor. Deciphering the pathways that are mediated specifically through the SFKs will add to our understanding of the physiological functions of even known and established GPCRs. Being able to attribute intercellular signals and subsequent cellular functions specifically to the SFK opens the opportunity for a so far untapped biased signaling approach that could be exploited by pharmaceutical interventions.

## Figures and Tables

**Table 1:** Overview of GPCRs that regulate or bind SFKs. Summarized are most of the GPCRs known to activate SFKs in a G protein- or arrestin-dependent manner. Some studies showed a direct interaction between the receptors and the SFKs, while in other studies, activation of the SFK was observed, but a mechanism was not defined.

GPCR	G-Protein	Arrestin	Direct	Other	References
α_2_AR		Arrestin-2/3			[42]
β_2_AR		Arrestin-2			[43,44]
β_3_AR			3rd ICL		[44]
D1R		Arresin-3		unknown	[45,46]
D2R		Arrestin-2		unknown	[47,48]
D4R			3rd ICL		[49]
V_1b_R				unknown	[50]
V_2_R			Indication of direct		[50]
GnRH-a	Indication of G_βγ_ protein				[51,52]
M1R	Indication of G_αq_ protein				[53]
M2R		Arrestin-2			[43,54]
M3R		Arrestin			[55]
M4R				unknown	[54]
B1R	G_αi_			unknown	[56,57]
ETAR				unknown	[58]
ATR2				unknown	[59,60]
Latrophilin-2				unknown	[61]
GPR56 (ADGRG1)				unknown	[62]

**Table 2:** Comparison of SFK binding motifs in the 3rd intracellular loop and C-tail of GPCRs. Shown are the amino acid Scheme 2. Y2 receptor, serotonin receptor type 6, and the subfamily of dopamine receptors, with highlighted polyproline motifs for SFKs in red and for other kinases in blue. Using a software to predict SH3 domain interactions, different SFK family members appeared to likely interact with individual domains [105].

	3ICL	C-Terminus	Predicted SFK SH3 Domain Interactions
**b1AR**	REAQKQVKKIDSCERRFLGG**P**AR**PP**SPSPSPVPA**P**A**PPP**G**PP**RPAAAAATAPLANGRAGKRRPSRLVALRE	CRSPDFRKAFQRLLCCARRAARRRHATHGDRPRASGCLAR**P**G**PPP**SPGAASDDDDDDVVGATPPARLLEPWAGCNGGAAADSDSSLDEPCRPGFASESKV	**FGR, LYN**
**b2AR**	RVFQEAKRQLQKIDKSEGRFHVQNLSQVEQDGRTGHGLRRSSKFCLKEHKALKT	PDFRIAFQELLCLRRSSLKAYGNGYSSNGNTGEQSGYHVEQEKENKLLCEDLPGTEDFVGHQGTVPSDNIDSQGRNCSTNDSLL	**-**
**b3AR**	RVFVVATRQLRLLRGELGRFPPEES**PP**A**P**SRSLAPAPVGTCAPPEGVPACGRRPARLLPLREHRALC	RSPDFRSAFRRLLCRCGRRL**PP**E**P**CAAARPALFPSGVPAARSS**P**AQ**P**RLCQRLDGASWGVS	**SRC, FGR, LYN, HCK, LCK, FYN**
**P2Y2**	MARRLLKPAYGTSGGLPRAKRKSVRT	GQRLVRFARDAKPPTGPSPATPARRRLGLRRSDRTDMQRIEDVLGSSEDSRRTESTPAGSENTKDIRL	**FGR**
**5HT6**	CRILLAARKQAVQVASLTTGMASQASETLQVPRTPRPGVESADSRRLATKHSRKALK	PLFMRDFKRALGRFLPC**P**RC**P**RERQASLASPSLRTSHSGPRPGLSLQQVLPLPLPPDSDSDSDAGSGGSSGLRLTAQLLLPGEATQD**PP**L**P**TRAAAAVNFFNIDPAEPELRPHPLGIPTN	**LYN**
**D1R**	RIAQKQIRRIAALERAAVHAKNCQTTTGNGKPVECSQPESSFKMSFKRETKVLK	RKAFSTLLGCYRLCPATNNAIETVSINNNGAAMFSSHHEPRGSISKECNLVYLIPHAVGSSEDLKKEEAAGIARPLEKLSPALSVILDYDTDVSLEKIQPITQNGQHPT	-
**D2R**	IVLRRRRKRVNTKRSSRAFRAHLRAPLKGNCTHPEDMKLCTVIMKSNGSFPVNRRRVEAARRAQELEMEMLSSTSPPERTRYS**P**I**PP**SHHQLTLPDPSHHGLHSTPDSPAKPEKNGHAKDHPKIAKIFEIQTMPNGKTRTSLKTMSRRKLSQQKEKKATQ	EFRKAFLKILHC	-
**D3R**	RIYVVLKQRRRKRILTRQNSQCNSVRPGFPQQTLSPDPAHLELKRYYSICQDTALGGPGFQERGGELKREEKTRNSLSPTIAPKLSLEVRKLSNGRLSTSLKLG**P**LQ**P**RGVPLREKKATQ	NIEFRKAFLKILSC	LYN
**D4R**	ATFRGLQRWEVARRAKLHGRA**P**RR**P**SG**P**G**PP**S**P**T**PP**A**P**RL**P**QD**P**CG**P**DCA**PP**A**P**GL**P**RG**P**CGPDCA**P**AA**P**SLPQDPCGPDCA**PP**A**P**GL**PP**DPCGSNCAPPDAVRAAAL**PP**QT**PP**QTRRRRRAKITGRERKAMR	NAEFRNVFRKALRACC	SRC, FGR, HCK, LYN,
**D5R**	RIYRIAQVQIRRISSLERAAEHAQSCRSSAACAPDTSLRASIKKETKVLK	FNADFQKVFAQLLGCSHFCSRTPVETVNISNELISYNQDIVFHKEIAAAYIHMMPNAVTPGNREVDNDEEEGPFDRMFQIYQTSPDGDPVAESVWELDCEGEISLDKITPFTPNGFH	-
